# The Economic Value of Non-professional Care: A Europe-Wide Analysis

**DOI:** 10.34172/ijhpm.2021.149

**Published:** 2021-10-30

**Authors:** Luz María Peña-Longobardo, Juan Oliva-Moreno

**Affiliations:** Faculty of Law and Social Sciences, University of Castilla-La Mancha, Toledo, Spain.

**Keywords:** Non-professional Care, Economic Valuation, Europe, Caregiving, Family Care, Informal Care

## Abstract

**Background:** This paper had two aims. Firstly, to provide a broader view of the profile of non-professional caregivers in Europe, and secondly, to estimate the economic value of the non-professional caregiving.

**Methods:** The European Quality of Life Survey 2016/2017, carried out by Eurofound, was used. The target population of the survey was adults who care for a relative or friend in a total of 33 European countries. The opportunity cost method was used to estimate the economic value of caregiving, in which two of the activities forgone were analysed: paid activities (restricted to caregivers who were employed), for which the average gross wage of each country was used; and unpaid activities, for which the minimum gross wage of each country was used.

**Results:** There were more than 76 million non-professional caregivers in Europe that provide care for a relative or friend. This figure represents 12.7% of the population in Europe. The estimated time devoted to non-professional care in Europe reached 72 301.5 million hours in 2016. Sharp differences were found among countries. The economic value of that time is estimated at 576 000 million of euros, which represented about 3.63% of Europe’s gross domestic product (GDP).

**Conclusion:** This study shows the very important number of resources dedicated to the non-professional care of dependent people and their economic valuation. These results may be helpful in prospective analyses estimating future needs on professional and non-professional and for designing of long-term care (LTC) policies in Europe.

## Background

**Key Messages**

** Implications for policy makers**

- Non-professional care represent a vast amount of resources in Europe. The abrupt substitution of non-professional care for professional resources would put great pressure on the sustainability of long-term care (LTC) systems across Europe.
- LTC systems in Europe present a high heterogeneity. Some countries have clearly focused on professional services while others are strongly supported on non-professional care. It would be advisable to establish an appropriate balance that reconciles the well-being of the people cared for and the carers.
- Effective reconciliation of employment with caregiving is needed in countries where a large number of working age caregivers are unemployed or homemakers.

** Implications for the public**

Governments should pay more attention to family caregiving, which is a valuable and essential resource for the maintenance of European long-term care (LTC) systems. Public policies should promote measures to give social prestige to professional and non-professional caregivers, and seek to achieve an appropriate balance between both types of care. Therefore, the information provided here would help policy-makers in each country when they are designing any strategy or policy related to LTC systems. It might also lead to think that, because of the high heterogeneity among LTC systems, it would be advisable to establish a proper balance between the two types of caregiving. In order to reduce the high opportunity cost that family caregivers burden and improve their well-being, and for buildings more solid LTC systems, governments should pay more attention to family caregiving.

All health insurance systems seek to reduce uncertainty among individuals facing the advent of a disease or injury, to distribute risks and to facilitate payment for the healthcare required in these situations. At the same time, health systems not only have to respect norms of social justice (equity), but also have to allocate resources efficiently, avoiding their overuse (moral hazard). These aspects have been widely studied in relation to healthcare systems,^1-5^ but they are also important for the long-term care (LTC) system.^6-8^

 LTC systems in European countries are currently one of the pillars of their Welfare States,^9^ although it is not possible to speak of a common model in terms of organisation, financing, benefits or conditions of access.^10,11^ These systems have been developed at different speeds, with Northern and Western European countries being pioneers and with subsequent development in Southern European countries.^12^ In the case of the countries of Eastern Europe differences are even greater. The welfare systems of these countries are marked by the fall of the socialist regimes and the changes in their social and political models. The social policies developed during the time of the transition of political systems and later and the factors that have influenced each country have resulted in a great heterogeneity in the characterization of their welfare systems, including the LTC system.^13-16^

 However, despite being very different, LTC systems will have to face common challenges in the next few decades.^17^ The continuing increase in longevity − the number of Europeans aged 80+ is going to rise from 4.9% in 2016 to 13% in 2070 − will translate into a more pronounced demographic ageing, which is expected to result in an increasing need for LTC in the nearly future. This is due not only to the positive relationship between age and dependency, but also to an expected partial replacement of non-professional care by professional services,^8^ referring such care as the care provided by qualified people including services such as home care, day care, nursing home, etc. In this sense, the projections of the European Commission suggest that public spending on LTC will grow significantly in the coming decades.^18^

 It is important to stress that LTC has the specific characteristic of being in a “fragmented territory” between the family, on the one hand, and the provision of professional services, financed publicly and privately, on the other. In all countries, non-professional care plays a very important role. However, its presence and its weight in total LTC care is more or less relevant, depending on the social structure, economic and sociocultural frameworks, institutional context and previous policies carried out in each country.^19-21^ Non-professional care, also called family care or informal care, is a heterogeneous service, being provided mainly by direct relatives of dependent people, aimed at enabling these people to perform the basic and instrumental activities of daily life. This concept of care is not easy to define and can vary among countries, in the same way that its definition has been enriched and nuanced over time.^22,23^

 There is evidence that the rights, training, support and social recognition of non-professional caregivers are very different throughout Europe.^23,24^ Despite the efforts made in previous studies, our lack of knowledge about the number of non-professional caregivers and their situation is still important.^25,26^ Existing literature suggests that both professional and non-professional care play important roles in LTC systems across Europe. Even though non-professional care is not limited to the care provided by family members, family is a key factor for providing, supervising and coordinating care.^27^ In fact, in the last decades there has been an intense debate on the relationship between social/care policies and family responsibilities and involvement in care.^21,27-29^ In this sense, it is very interesting the characterization made by Leitner^29^ and continuing by other authors^30-32^ of different policies that lead to increasing the care responsibilities of family members (explicit or implicit familialism according to Leitner’s terminology), that weaken the responsibility of families (de-familialism) or that favour the right to care but without it having the character of obligation (optional familialism). The combination of time rights, cash for care and in-kind services present in a certain country, jointly with social structure and economic and sociocultural frameworks will determine the greater or lesser degree of responsibility of the family. To the above, it must be added that demographic trends now indicate a strong increase in the older population in the coming decades and a reduction in the capacity of family networks to provide support, especially that of middle-aged women.^33-36^ These trends point towards an increase in the number of working women, a decreased family size and increases in the retirement age. In this sense, those LTC models where the responsibility for the care of dependent people falls excessively on the family do not seem to be the most appropriate one when it is consideredthat a high intensity of care can cause a worsening of the state of health, problems in the workplace and in social and family relationships.^37-40^

 An economic analysis of the role of caregiving provided by non-professional caregivers, mainly relatives and friends, would then enable one to draw attention to the value of this social resource within LTC systems.^35,41-44^ Hence, even though there are a wide range of studies which have analysed the cost associated with non-professional caregivers in Europe,^45-50^ most of them are focused on a specific chronic disease or a specific country. So this work tries to fill some of the existing gaps in information about non-professional care in Europe. First, it would provide an estimation of the number and a wider view of the profile of non-professional caregivers in Europe, and secondly, it would enable us to estimate the economic value of non-professional time care, in monetary terms and in relation to gross domestic product (GDP), analysing the differences among the European countries.

## Methods

###  Data

 The European Quality of Life Survey 2016, carried out by Eurofound between September 2016 and March 2017, was used. It contains information about a range of subjects, such as employment, income, education, housing, family, careers, health and work/life balance. It also looks at subjective topics, such as people’s levels of happiness, how satisfied they are with their lives, and how they perceive the quality of their societies. The target population of the survey was adult residents (over 18 years of age) from a total of 33 European countries. Through a personal interview system, the respondents answered questionnaires in their own homes about the various topics of interest. In order to collect information about non-professional caregiving, the interviewees were then asked how much time they spent caring for relatives, neighbours or dependent friends during the previous week. Additionally, the survey examined representative national and international samples of the disabled population, so that, through the weights given, the figures obtained could be extrapolated to the total disabled population living in all of Europe and in each country included.

 This survey was chosen to analyse the characteristics and the economic value of non-professional caregiving in Europe because up to the date of carrying out this work, it was the survey that contained the most up-to-date information about the number of hours of non-professional caregiving in all of Europe. Another reason for choosing it was that it contained information about all adult caregivers, not only those older than 50, as in other European surveys, such as the Survey of Health, Ageing and Retirement in Europe-SHARE.

###  Main Variable

 In order to collect information about non-professional caregiving, the interviewees were then asked how much time they spent caring for relatives, neighbours or dependent friends during the previous week. Individuals who responded “more than 0 hours” to this question, were identified as non-professional caregivers. Table S1 in Supplementary file 1 describes all variables of interest.

###  The Technique Used to Estimate the Economic Value of Non-professional Caregiving

 The opportunity cost technique was applied. This is one of the most frequently used methods in the literature.^48-53^ It values the non-professional caregiving time provided by taking into account the benefits forgone by the caregiver due to the tasks provided. In other words, this method values the best alternatives that caregivers had to forgo in order to provide the care.^22,53^

 In general, the sacrificed time (that is, the benefits forgone) includes paid work time, unpaid work time (such as housekeeping or voluntary work), and leisure time. Thus, the shadow price applied in each activity depends on the type of activity forgone. More precisely, two forgone activities were analysed in terms of time: paid work time (for those caregivers who were employed) and unpaid time (for those caregivers who were not employed). First, to value paid work time, the average gross hourly wage in purchasing power parity in each country included in the analysis (Eurostat data) was used, taking into account the caregiving hours provided by those caregivers who were employed. In the case of Albania, where the information about average hourly wage was not available, the shadow price of the FYR (Former Yugoslav Republic) of Macedonia was used (due to geographical and cultural similarities). This was done by adjusting its GDP per capita to that of the FYR of Macedonia. Secondly, to value unpaid work time, the number of caregiving hours provided by those caregivers who were not in the labour market because they were retired or were dedicated to housework tasks was considered. This time was valued using the minimum gross monthly wage in each country in purchasing power parity (Eurostat data), which was then converted to the minimum gross hourly wage taking into account the weekly average number of working days in each country, according to Eurostat. When the data about minimum wage were not available (as was the case with Denmark, Italy, Cyprus, Austria, Finland and Sweden), the proportion that the minimum gross wage represents in relation to the average gross wage in countries with geographical and cultural similarities was used. In our main analysis we employed average wages. For the purpose to develop an alternative estimation that can be used as a sensitivity analysis, we also employed median wages (results are shown in Supplementary file 1).

 In brief, the specification of the opportunity cost of non-professional caregiving was as follows:


$$C_{OPP}=\sum_{i=1}^{n}\sum_{j=1}^{33}\left(np_{i}W_{j}+na_{i}S_{j}\right)$$

where *C*_OPP_ represents the total annual opportunity cost of non-professional care in euros; *npi* is the caregiving hours provided by the caregiver *i* who was employed; *Wj* is the average hourly wage in each country *j*; *nai* is the caregiving hours provided by the caregiver *i* who was retired or unemployed or dedicated to housework tasks or a student; and *Sj* is the minimum hourly wage in each country *j*.

 All the costs were expressed in 2016 euros. Additionally, we use data on public spending on LTC in each country in proportion to its GDP. The purpose of including this information is to have a reference figure to compare the estimated on the aggregate economic value of non-professional care in each country with the effort made in the public policies of professional LTC.

## Results

###  Profile of Non-professional Caregivers

 The estimation of non-professional caregivers in Europe exceeds 76 million people. This means that 12.7% of the population in Europe provide care for a relative or friend. The majority of them, 61.4%, are women with an average age of 52. About 25% have had higher education while 36% have had lower or below secondary education. 50% combine paid work with caregiving, almost 8% are unemployed, 2% are unable to work, 23.6% are retired, 11.3% are full-time homemakers, 3.4% are students and 0.4% are in other situations (Table 1).

**Table 1:** Profile of Non-professional Caregivers by Country

	**Number of Caregivers** **No. (%)**	**Female** **No. (%) **	**Age Mean (SD) **	**High Level of Education ** **No. (%)**	**Upper Secondary or Post-secondary Education** **No. (%)**	**Lower Secondary Education or Below ** **No. (%)**	**Employed ** **No. (%)**	** Unemployed ** **No. (%)**	**Unable to Work** **No. (%)**	**Retired** **No. (%)**	**Full-time Homemaker** **No. (%)**	**Student** **No. (%)**	**Other Employment Situation** **No. (%)**
Albania	421 150 (14.65)	273 729 (65.00)	49.21 (15.22)	70 495 (16.74)	139 575 (33.14)	211 080 (50.12)	101 989 (24.22)	114 384 (27.16)	11 596 (2.75)	74 375 (17.66)	97 091 (23.05)	21 715 (5.16)	-
Austria	635 477 (7.30)	424 883 (66.86)	52.46 (12.82)	292 628 (46.05)	313 574 (49.34)	29 275 (4.61)	349 430 (54.99)	45 863 (7.22)	12 804 (2.01)	198 694 (31.27)	26 152 (4.12)	2534 (0.40)	-
Belgium	2 471 206 (21.85)	1 489 300 (60.27)	52.79 (16.86)	826 730 (33.45)	1 040 309 (42.10)	604 167 (24.45)	1 174 577 (47.53)	197 942 (8.01)	130 323 (5.27)	600 235 (24.29)	98 993 (4.01)	194 960 (7.89)	74 176 (3.00)
Bulgaria	550 189 (7.69)	343 490 (62.43)	55.15 (14.14)	125 499 (22.81)	217 927 (39.61)	206 763 (37.58)	223 079 (40.55)	67 064 (12.19)	6227 (1.13)	214 906 (39.06)	27 286 (4.96)	11 627 (2.11)	-
Croatia	532 633 (12.71)	361 926 (67.95)	49.45 (13.29)	111 188 (20.88)	374 873 (70.38)	46 572 (8.74)	274 708 (51.58)	66 478 (12.48)	-	128 085 (24.05)	56 536 (10.61)	6826 (1.28)	-
Cyprus	95 130 (11.21)	63 136 (66.37)	53.63 (16.09)	29 648 (31.17)	29 212 (30.71)	36 270 (38.13)	37 887 (39.83)	15 200 (15.98)	1653 (1.74)	23 719 (24.93)	12 886 (13.55)	2226 (2.34)	1559 (1.64)
Czech Republic	1 182 716 (11.21)	772 869 (65.35)	51.55 (15.06)	148 435 (12.55)	915 086 (77.37)	119 195 (10.08)	697 806 (59.00)	83 210 (7.04)	16 749 (1.42)	276 396 (23.37)	26 395 (2.23)	61 123 (5.17)	21 037 (1.78)
Denmark	713 597 (12.50)	429 472 (60.18)	56.51 (14.33)	225 075 (31.54)	340 556 (47.72)	147 966 (20.74)	437 110 (61.25)	29 822 (4.18)	22 034 (3.09)	199 577 (27.97)	-	18 582 (2.60)	6472 (0.91)
Estonia	150 636 (11.45)	104 230 (69.19)	56.24 (16.61)	44 222 (29.36)	81 282 (53.96)	25 132 (16.68)	84 397 (56.03)	4597 (3.05)	10 209 (6.78)	42 296 (28.08)	2928 (1.94)	5045 (3.35)	1164 (0.77)
Finland	1 044 290 (19.03)	577 398 (55.29)	59.00 (13.15)	438 031 (41.95)	385 556 (36.92)	220 703 (21.13)	522 865 (50.07)	29 564 (2.83)	15 686 (1.50)	434 236 (41.58)	14 188 (1.36)	21 719 (2.08)	6032 (0.58)
France	15 406 358 (23.12)	9 316 186 (60.47)	49.76 (16.14)	5 699 542 (36.99)	3 813 000 (24.75)	5 893 816 (38.26)	8 814 928 (57.22)	1 047 716 (6.80)	478 882 (3.11)	3 952 091 (25.65)	646 994 (4.20)	465 747 (3.02)	-
FYR of Macedonia	156 744 (7.57)	71 388 (45.54)	47.65 (15.02)	36 104 (23.03)	79 198 (50.53)	41 442 (26.44)	57 702 (36.81)	58 713 (37.46)	2 087 (1.33)	11 529 (7.36)	24 765 (15.80)	1948 (1.24)	-
Germany	5 224 257 (6.36)	2 900 996 (55.53)	52.49 (15.16)	1 197 486 (22.92)	1 849 102 (35.39)	2 177 669 (41.68)	3 236 706 (61.96)	231 434 (4.43)	104 870 (2.01)	1 101 721 (21.09)	260 644 (4.99)	288 882 (5.53)	-
Greece	1 213 335 (11.25)	837 436 (69.02)	54.90 (14.29)	288 185 (23.75)	524 072 (43.19)	401 078 (33.06)	444 608 (36.64)	259 187 (21.36)	-	215 850 (17.79)	277 143 (22.84)	16 547 (1.36)	-
Hungary	773 751 (7.87)	447 282 (57.81)	51.15 (14.14)	186 508 (24.10)	144 826 (18.72)	442 417 (57.18)	414 938 (53.63)	98 209 (12.69)	11 994 (1.55)	207 815 (26.86)	37 213 (4.81)	3582 (0.46)	-
Ireland	588 522 (12.45)	343 417 (58.35)	51.76 (15.88)	202 837 (34.47)	241 522 (41.04)	144 163 (24.50)	220 915 (37.54)	36 942 (6.28)	9210 (1.56)	131 690 (22.38)	163 306 (27.75)	26 459 (4.50)	-
Italy	7 959 719 (13.12)	5 277 486 (66.30)	54.02 (14.23)	1 201 416 (15.09)	3 724 014 (46.79)	3 034 289 (38.12)	3 748 365 (47.09)	546 393 (6.86)	35 266 (0.44)	2 059 512 (25.87)	1 427 990 (17.94)	142 193 (1.79)	-
Latvia	416 678 (21.16)	261 123 (62.67)	54.77 (15.89)	114 775 (27.55)	255 686 (61.36)	46 217 (11.09)	221 447 (53.15)	38 859 (9.33)	10 114 (2.43)	125 449 (30.11)	14 268 (3.42)	6541 (1.57)	-
Lithuania	336 016 (11.63)	232 707 (69.25)	53.18 (15.79)	133 289 (39.67)	167 661 (49.90)	35 066 (10.44)	191 440 (56.97)	40 748 (12.13)	20 348 (6.06)	68 559 (20.40)	7200 (2.14)	7721 (2.30)	-
Luxembourg	97 824 (16.98)	57 835 (59.12)	49.22 (14.12)	33 505 (34.25)	39 887 (40.77)	24 432 (24.98)	60 934 (62.29)	4182 (4.28)	2492 (2.55)	22 327 (22.82)	5704 (5.83)	2022 (2.07)	163 (0.17)
Malta	68 992 (15.32)	47 063 (68.22)	55.73 (15.16)	10 979 (15.91)	25 840 (37.45)	32 173 (46.63)	22 774 (33.01)	1393 (2.02)	560 (0.81)	14 210 (20.60)	26 461 (38.35)	2751 (3.99)	843 (1.22)
Montenegro	71 422 (11.48)	35 352 (49.50)	41.49 (13.87)	15 866 (22.21)	53 346 (74.69)	2210 (3.09)	39 416 (55.19)	14 281 (20.00)	-	8754 (12.26)	2339 (3.27)	6632 (9.29)	-
Netherlands	2 758 459 (16.25)	1 667 001 (60.43)	55.72 (13.41)	925 132 (33.54)	925 558 (33.55)	907 769 (32.91)	1 416 818 (51.36)	142 727 (5.17)	192 222 (6.97)	755 392 (27.38)	210 731 (7.64)	25 518 (0.93)	15 051 (0.55)
Poland	4 007 885 (10.56)	3 005 338 (74.99)	52.02 (13.29)	479 311 (11.96)	3 147 817 (78.54)	380 757 (9.50)	1 867 363 (46.59)	347 156 (8.66)	216 454 (5.40)	1 158 288 (28.90)	289 260 (7.22)	65 425 (1.63)	63 939 (1.60)
Portugal	599 691 (5.80)	328 789 (54.83)	56.38 (14.11)	126 841 (21.15)	104 709 (17.46)	368 141 (61.39)	331 084 (55.21)	45 677 (7.62)	-	165 361 (27.57)	57 569 (9.60)	-	-
Roumania	2 573 923 (13.03)	1 635 509 (63.54)	50.66 (14.57)	209 765 (8.15)	1 521 223 (59.10)	842 935 (32.75)	1 149 174 (44.65)	14 505 (0.56)	-	646 540 (25.12)	657 623 (25.55)	73 346 (2.85)	32 735 (1.27)
Serbia	1 242 649 (17.56)	711 874 (57.29)	49.67 (14.89)	139 530 (11.23)	925 675 (74.49)	177 444 (14.28)	525 789 (42.31)	272 029 (21.89)	14 518 (1.17)	276 432 (22.25)	110 066 (8.86)	43 815 (3.53)	-
Slovakia	487 899 (8.99)	315 981 (64.76)	54.87 (13.21)	74 399 (15.25)	221 584 (45.42)	191 916 (39.34)	260 713 (53.44)	29 117 (5.97)	-	153 107 (31.38)	4632 (0.95)	28 865 (5.92)	11 465 (2.35)
Slovenia	272 813 (13.22)	166 249 (60.94)	50.42 (15.12)	71 746 (26.30)	101 135 (37.07)	99 932 (36.63)	150 350 (55.11)	20 922 (7.67)	-	84 844 (31.10)	4952 (1.82)	11 745 (4.31)	-
Spain	6 086 020 (13.11)	4 078 673 (67.02)	49.59 (14.91)	1 162 050 (19.09)	2 483 673 (40.81)	2 440 297 (40.10)	2 375 172 (39.03)	923 710 (15.18)	159 756 (2.62)	938 123 (15.41)	1 228 226 (20.18)	433 378 (7.12)	27 655 (0.45)
Sweden	976 420 (9.91)	546 534 (55.97)	60.30 (14.96)	411 773 (42.17)	399 977 (40.96)	164 670 (16.86)	509 522 (52.18)	8971 (0.92)	28 362 (2.90)	420 989 (43.12)	-	8576 (0.88)	-
Turkey	7 744 004 (9.83)	4 362 550 (56.33)	38.43 (14.58)	1 092 127 (14.10)	2 511 922 (32.44)	4 139 955 (53.46)	3 438 901 (44.41)	500 154 (6.46)	-	905 302 (11.69)	2 311 058 (29.84)	588 589 (7.60)	-
United Kingdom	9 312 086 (14.24)	5 317 414 (57.10)	51.49 (16.23)	2 789 989 (29.96)	2 762 639 (29.67)	3 759 458 (40.37)	5 173 031 (55.55)	707 582 (7.60)	359 403 (3.86)	2 365 881 (25.41)	519 684 (5.58)	102 737 (1.10)	83 768 (0.90)
**Total**	**76 172 491 (12.70)**	**46 804 616 (61.45) **	**51.97 (15.53)**	** 18 915 106 (24.83) **	** 29 862 016 (39.20) **	** 27 395 369 (35.96) **	** 38 575 938 (50.64) **	** 6 044 731** **(7.94) **	** 1 873 819 (2.46) **	** 17 982 285 (23.61) **	** 8 650 283 (11.36) **	** 2 699 376 (3.54) **	** 346 059 (0.45) **

Abbreviations: SD, standard deviation; FYR, Former Yugoslav Republic. Source: Own preparation. All sample data have been extrapolated to population level.

 The profile of a non-professional caregiver in Europe differs significantly depending on the country. France, Belgium, Finland and Serbia are the countries where the proportions of non-professional caregivers in relation to their populations are the highest (23%, 22%, 19% and 17% respectively), while in countries such as Germany or Austria this percentage reaches 6% and 7% respectively. Regarding age, in most of the countries non-professional caregivers are between 48 and 53 years old, except in Turkey, where caregivers are significantly younger than in the other countries (with an average of 38.4 years old). Another difference found in caregivers in Europe is related to their level of education. Generally speaking, non-professional caregivers in Northern European countries, such as Sweden and Finland, and in other countries of Central Europe such as Austria, are well educated, as 42%, 42% and 46% of them respectively have completed their tertiary education, while in countries such as Poland and Romania, these figures barely reach 12% and 8% respectively. Lower or below secondary education is highlighted in Portugal, Hungary and Albania, where the percentages of non-professional caregivers with that level of education reach 61%, 57% and 50% respectively. Differences were also found in relation to their employment situation. More than 62% in Luxembourg and Germany are employed, while only 24% and 36%, respectively, are employed in Albania and Greece. The highest rates of unemployment were in the FYR of Macedonia and in Albania, with 37% and 27% of non-professional caregivers not having a job, respectively. Meanwhile, more than 43% and 41% of the caregivers are retired in Sweden and Finland, respectively, and the countries with the largest numbers of non-professional caregivers as full-time homemakers are Malta and Turkey with 38% and 30%, respectively.

 Regarding their health-related quality of life, about 7% of caregivers consider that their health is “bad or very bad,” with an average of 6.68 (out of 10) being “satisfied” with their lives, and 22% of them stating that it is “quite difficult” to combine paid work with care responsibilities. 10.6% state that they are “quite satisfied” with the quality of LTC services, with a score of 7.32 (out of 10), although about 10% of them consider that it is “difficult” to cover costs related to LTC (Table 2). Differences among countries were also found in this area. Regarding the health-related quality of life of caregivers, those in Albania, Estonia, Lithuania are the ones with the worst state of health, with more than 18%, 17% and 16% of them respectively declaring that their health is “bad or very bad.” In contrast, these figures hardly reach 2% in countries such as Malta, Luxembourg, Ireland and France. Likewise, the life satisfaction of the caregivers differs a lot among countries. Thus, Northern European countries such as Denmark, Finland, Sweden and the Netherlands are the countries whose caregivers are the most satisfied with their lives, with scores of 8.33, 8.04, 7.88 and 7.74 points respectively (out of 10). Countries such as Austria, Ireland and Malta also have similar scores. In contrast, Albania, the FYR of Macedonia and Greece are those with the lowest scores in life satisfaction, with 5.54, 4.26 and 4.64 points respectively (Table 2).

**Table 2:** Health and Long-term Care-Related Characteristics of Non-professional Caregivers by Country

	**Bad or Very Bad HRQoL** **No. (%)**	**Life Satisfaction ** **Mean (SD)** ^a^	**Satisfaction with LTC** **Mean (SD)** ^a^	**Difficulties to Combine Work and Caregiving** ^b^ **No. (%)**	**Cost Difficulty in LTC (a Little or Very Difficult) ** **No. (%)**
Albania	77 306 (18.36)	4.57 (2.84)	7.00 (2.40)	59 586 (14.15)	44 213 (10.50)
Austria	47 213 (7.43)	7.70 (2.17)	7.35 (2.16)	75 617 (11.90)	166 341 (26.18)
Belgium	145 573 (5.89)	7.15 (1.95)	7.82 (1.41)	37 710 (1.53)	321 115 (12.99)
Bulgaria	47 508 (8.63)	5.13 (2.27)	6.00 (0.00)	168 282 (30.59)	29 074 (5.28)
Croatia	69 991 (13.14)	6.41 (2.11)	6.83 (3.18)	145 575 (27.33)	32 793 (6.16)
Cyprus	10 097 (10.61)	5.93 (2.47)	7.2 (1.48)	22 185 (23.32)	23 642 (24.85)
Czech Republic	88 248 (7.46)	6.20 (2.05)	6.6 (1.89)	432 775 (36.59)	196 729 (16.63)
Denmark	102 629 (14.38)	8.33 (1.69)	8.59 (1.77)	89 479 (12.54)	24 140 (3.38)
Estonia	26 442 (17.55)	6.25 (2.09)	8.00 (1.78)	18 527 (12.30)	18 091 (12.01)
Finland	42 403 (4.06)	8.04 (1.28)	7.84 (1.71)	124 942 (11.96)	120 754 (11.56)
France	435 735 (2.83)	6.95 (1.87)	7.12 (2.22)	2 734 438 (17.75)	819 784 (5.32)
FYR of Macedonia	11 200 (7.15)	4.26 (2.40)	4.37 (1.40)	17 391 (11.10)	34 087 (21.75)
Germany	252 775 (4.84)	7.17 (1.96)	7.82 (2.15)	1 211 458 (23.19)	307 419 (5.88)
Greece	76 100 (6.27)	4.64 (2.41)	6.57 (3.04)	212 284 (17.50)	144 862 (11.94)
Hungary	99 608 (12.87)	6.51 (2.12)	7.71 (2.21)	182 947 (23.64)	75 337 (9.74)
Ireland	12 264 (2.08)	7.50 (1.59)	7.82 (2.18)	45 818 (7.79)	92 525 (15.72)
Italy	334 436 (4.20)	6.39 (1.69)	6.34 (1.98)	1 742 935 (21.90)	1 292 699 (16.24)
Latvia	57 969 (13.91)	5.93 (2.00)	7.21 (2.46)	61 373 (14.73)	44 563 (10.69)
Lithuania	54 981 (16.36)	6.14 (2.28)	8.28 (0.95)	45 213 (13.46)	27 380 (8.15)
Luxembourg	2730 (2.79)	7.88 (1.82)	8.55 (1.84)	21 220 (21.69)	11 187 (11.44)
Malta	1 973 (2.86)	7.58 (1.82)	8.3 (1.05)	11 770 (17.06)	2285 (3.31)
Montenegro	5 063 (7.09)	5.99 (2.47)	5.76 (2.65)	15 501 (21.70)	16 141 (22.60)
Netherlands	245 825 (8.91)	7.74 (1.31)	7.71 (1.23)	307 881 (11.16)	207 713 (7.53)
Poland	434 088 (10.83)	6.69 (2.21)	6.66 (1.75)	889 193 (22.19)	220 300 (5.50)
Portugal	52 955 (8.83)	6.95 (1.88)	7.33 (0.57)	31 356 (5.23)	49 685 (8.29)
Roumania	438 192 (17.02)	6.06 (2.42)	9.20 (0.83)	524 917 (20.39)	51 319 (1.99)
Serbia	133 351 (10.73)	6.02 (2.30)	7.54 (2.01)	347 742 (27.98)	124 069 (9.98)
Slovakia	47 010 (9.64)	6.07 (2.36)	6.80 (2.94)	117 362 (24.05)	36 531 (7.49)
Slovenia	28 623 (10.49)	7.24 (1.94)	8.11 (2.05)	52 949 (19.41)	24 019 (8.80)
Spain	183 069 (3.01)	6.72 (2.16)	7.75 (1.13)	1 235 301 (20.30)	341 392 (5.61)
Sweden	115 783 (11.86)	7.88 (1.73)	7.65 (2.20)	153 197 (15.69)	59 043 (6.05)
Turkey	525 136 (6.78)	5.64 (2.13)	6.25 (2.14)	1 433 167 (18.51)	2 472 506 (31.93)
United Kingdom	917 032 (9.85)	7.47 (1.90)	6.31 (2.90)	1 793 346 (19.26)	623 980 (6.70)
**Total**	** 5 123 308 (6.73) **	**6.68 (2.50)**	**7.32 (2.14)**	** 14 363 437 (18.86) **	**8 055 718 (10.58)**

Abbreviations: HRQoL, Health-related quality of life; SD, standard deviation; FYR, Former Yugoslav Republic; LTC, long-term care. ^a^Mean (SD)“10” very satisfied and “1” very dissatisfied.^b^How easy or difficult is it to combine paid work with your care responsibilities? (rather or very difficult). Source: own elaboration. All sample data have been extrapolated to population level.

 Another interesting point is the satisfaction that caregivers have with LTC facilities. In this area, Romanian caregivers are the most satisfied, with an average score of 9.2 points (out of 10), followed by caregivers in Denmark, Luxembourg and Lithuania, with 8.59, 8.55, 8.28 points respectively. In contrast, Montenegro, Turkey and the United Kingdom have the lowest rate of satisfaction with LTC facilities, with an average of 5.76, 6.25 and 6.31 points respectively. Finally, the countries in which caregivers find it “quite difficult” to combine work and caregiving tasks are Bulgaria and Croatia, with about 30% of them stating this (Table 2).

###  The Economic Value of Non-professional Caregiving

 In Europe, more than 72 000 million non-professional caregiving hours are provided annually (18.25 weekly hours per caregiver) (Tables 3 and 4). This figure would represent about 3.64% of the GDP in Europe. On average, the value of the care provided by each non-professional caregiver is estimated at €7567 annually. Again, important differences are found when analysing by country. In terms of intensive caregiving, the cases of Turkey, France, the United Kingdom, Spain and Italy are notable, with more than 13 000, 11 000, 9000, 6600 and 6000 million caregiving hours annually, respectively. This scenario changes slightly when considering the average hours of caring per caregiver. Thus, Turkey, Bulgaria, Poland and Ireland are the countries where the intensity of caregiving per caregiver is the highest, with 32.50, 23.72, 27.30 and 23.50 weekly hours respectively. In contrast, the intensity of caregiving is significantly lower in countries such as Finland, Sweden and Denmark, with 8.34, 8.26 and 6.47 weekly hours respectively (Table 3).

**Table 3:** Non-professional Hours Provided and Value of Non-professional Care by Country, Euros in 2016

	**Average Weekly Caregiving Hours Per Carer**	**Aggregated Annual Number of Caregiving Hours**	**Aggregated Annual Number of Caregiving Hours (Employed Carers)**	**Aggregated Annual Number of Caregiving Hours (Non-employed Carers)**	**Aggregated Annual Non-professional Caregiving Value (€)**	**Annual non-Professional Caregiving Value Per Carer (€)**
Albania	23.83	521 924 494	68 223 800	453 700 694	638 383 389	1515
Austria	18.93	625 660 548	215 130 960	410 529 588	6 803 917 472	10 706
Belgium	11.24	1 444 434 065	503 033 427	941 400 638	18 858 771 568	7631
Bulgaria	23.72	678 554 592	153 233 992	525 320 600	1 059 251 801	1925
Croatia	15.86	439 167 015	147 310 482	291 856 533	1 574 413 123	2955
Cyprus	17.58	86 963 904	21 778 299	65 185 605	526 122 457	5530
Czech Republic	18.96	1 166 000 000	320 042 194	845 957 806	3 621 959 164	3062
Denmark	6.47	240 011 156	116 476 360	123 534 796	4 939 505 716	6921
Estonia	11.20	87 736 306	34 890 540	52 845 766	347 678 215	2308
France	14.04	11 248 236 380	5 024 000 000	6 224 236 380	145 223 956 505	9426
FYR of Macedonia	19.00	154 843 920	32 223 438	122 620 482	254 832 200	1625
Germany	10.69	2 904 100 000	1 549 400 000	1 354 700 000	40 165 022 155	7688
Greece	20.10	1 268 022 168	227 951 858	1 040 070 310	6 134 557 811	5055
Finland	8.34	452 825 100	161 442 762	291 382 338	6 107 972 858	5848
Hungary	18.60	748 549 170	196 310 523	552 238 647	2 133 948 806	2757
Italy	14.80	6 126 800 000	1 832 700 000	4 294 100 000	55 076 278 850	6919
Ireland	23.05	705 463 616	127 119 069	578 344 547	8 829 221 918	15 002
Lithuania	18.51	323 495 078	137 794 740	185 700 338	957 260 409	2848
Luxembourg	14.70	74 752 420	49 065 677	25 686 743	1 438 251 518	14 702
Latvia	16.73	362 391 678	114 150 001	248 241 677	1 074 706 056	2579
Malta	21.70	77 860 224	16 471 915	61 388 309	439 049 362	6363
Netherlands	10.41	1 492 948 170	519 476 310	973 471 860	18 479 678 120	6699
Poland	27.30	5 688 900 000	1 128 900 000	4 560 000 000	18 207 393 633	4542
Portugal	13.25	413 284 040	202 532 628	210 751 412	2 339 607 698	3901
Roumania	26.13	3 498 000 000	1 112 800 000	2 385 200 000	6 689 686 066	2599
Slovakia	20.35	516 250 868	182 229 164	334 021 704	1 781 140 583	3650
Montenegro	21.74	80 759 647	32 191 766	48 567 881	220 068 540	3081
Serbia	14.31	924 513 398	314 191 354	610 322 044	1 812 120 746	1458
Sweden	8.26	419 231 694	105 910 945	313 320 749	5 590 618 637	5725
Slovenia	16.36	232 034 005	97 327 377	134 706 628	1 518 767 032	5567
Spain	21.13	6 685 900.000	1 859 200 000	4 826 700 000	44 611 163 501	7330
Turkey	32.50	13 086 300 000	5 103 000 000	7 983 300 000	43 537 057 304	5622
United Kingdom	19.67	9 525 600 000	3 955 600 000	5 570 000 000	125 440 377 094	13 470
**Total**	**18.25**	**72 301 513 656**	**25 662 109 581**	**46 639 404 075**	**576 432 740 322 **	**7567**

Abbreviations: SD, standard deviation; FYR, Former Yugoslav Republic. Source: own elaboration. All sample data have been extrapolated to population level.

 In terms of the estimated value, France, the United Kingdom, Italy, Spain, Turkey and Germany are the countries with the highest economic value of non-professional caregiving, with 145 200, 125 400, 55 100, 44 600, 43 500 and 40 200 million euros annually. These figures are equivalent to 6.50%, 5.15%, 3.25%, 4.0%, 5.56% and 1.28% of their GDPs, respectively. If we take into account the cost per caregiver, Ireland is the country with the highest average value per carer (€15 000), followed by Luxembourg (€14 702), the United Kingdom (€13 479), Austria (€10 706) and France (€9426). Conversely, countries such as Serbia, Albania and the FYR of Macedonia have the lowest average value, €1458, €1515 and €1458, respectively (Table 4). Table S2 in Supplementary file 1 shows the results applying the median wages instead of average wages, in which is observed a total cost of €516 335 million of euros (with an average cost of €6778 per caregiver).

**Table 4:** Value of Non-professional Care by Country, Habitants, Long-term Care Expenditure and Gross Domestic Product, Euros in 2016

	**Annual Non-professional Caregiving Value/Habitants >65 (€)**	**Annual Non-professional Caregiving Value/Habitants (€)**	**% Annual Non-professional Caregiving Value/GDP**	**% Public LTC Expenditure/GDP**
Albania	1735.18	222.00	5.96	-
Austria	4239.29	782.02	1.90	1.9
Belgium	9141.86	1667.28	4.38	2.3
Bulgaria	724.63	148.07	2.18	0.4
Croatia	1956.07	375.69	3.38	0.9
Cyprus	4102.64	620.19	2.79	0.3
Czech Republic	1874.32	343.19	2.05	1.3
Denmark	4597.36	865.48	1.74	2.5
Estonia	1388.91	264.20	1.60	0.9
France	11 556.20	2179.28	6.50	1.7
FYR of Macedonia	947.34	123.03	2.64	-
Germany	2321.65	488.77	1.28	1.3
Greece	2675.51	568.87	3.48	0.1
Finland	5438.48	1113.11	2.81	2.2
Hungary	1187.78	217.07	1.85	0.7
Italy	4119.47	907.87	3.25	1.7
Ireland	14 137.64	1868.11	3.25	1.3
Lithuania	1745.15	331.40	2.46	1.0
Luxembourg	17 548.86	2495.89	2.62	1.3
Latvia	2780.00	545.83	4.29	0.4
Malta	5269.75	974.77	4.25	0.9
Netherlands	5989.57	1088.38	2.61	3.5
Poland	3004.78	479.56	4.27	0.5
Portugal	1092.85	226.24	1.25	0.5
Roumania	1947.25	338.54	3.93	0.3
Slovakia	2272.05	328.25	2.20	0.9
Montenegro	2514.58	353.68	5.57	-
Serbia	1348.89	256.08	4.93	-
Sweden	2871.07	567.52	1.20	3.2
Slovenia	3995.68	735.77	3.76	0.9
Spain	5131.16	960.62	4.01	0.9
Turkey	6702.92	552.91	5.58	-
United Kingdom	10 709.87	1918.66	5.15	1.5
**Total**	**5422.89**	**958.22**	**3.64**	**-**

Abbreviations: GDP, gross domestic product; FYR, Former Yugoslav Republic; LTC, long-term care.

 When comparing the estimated value for the total population with those for the population older than 65 years in each country, different figures are shown. Broadly, the value estimated for non-professional caregiving stands at €958 per inhabitant in all Europe. However, this figure is considerably higher when taking into account the older population (older than 65 years), where the value of non-professional care reaches €5422 per inhabitant (Table 4).

## Discussion

 Generally speaking, this paper covers a broader perspective in the field of non-professional care, being the first one that provides information about the economic value (opportunity cost) of non-professional caregiving in 33 different European countries. Furthermore, given the data set used, it has been possible to consider all European adults who provide care, not only those older than 50, even though other studies and analyses have only focussed on older population.^34,35^ The results estimate that more than 72 000 million hours of non-professional care are provided in the 33 Europe countries examined in this study and that almost 13% of the population in Europe (76 million of inhabitants) are involved in the provision of care for a relative or friend, with a predominant role for middle-aged women.

 The results also reveal differences between countries that deserve to be highlighted. There are countries, such as France and Belgium, where a high percentage of caregiver population is reported, in relation to the total population (around 23%). In other countries, such as Turkey, Bulgaria, Poland and Ireland, the high intensity of attention, measured in number of hours, stands out, while in the countries of northern Europe, the intensity is notably lower.

 There are certain patterns to be expected ex ante that are present in some countries. For instance, the presence of non-professional caregiving is very high (not only in terms of intensity but also in terms of opportunity cost between 4% and 5% of their GDP) in countries, such as Turkey, Ireland, Spain, Italy, Greece and the Eastern European, where the effort in terms of public LTC expenditure is quite low (about 0.5%-1% of their GDP). In contrast, several countries such as Germany, Austria, the Netherlands and the Northern European countries (Sweden, Denmark and Finland), perform considerable efforts in LTC public spending (about 2% of their GDP), being the presence of non-professional care quite low. However, for the complete set of countries analysed, there is no significant association between LTC public spending and the amount of non-professional care provided. This could be a reflection of the great differences that exist in the organisational forms and structures of LTC systems in Europe, but also of the complexity of the interrelationships that exist between professional and non-professional care, mainly explained by social and cultural norms of each country and for the inheritance of previous policies. In this sense, it is worth noting that the same policy can have very different effects depending on the rest of the elements present in the country. For example, cash for care policies can favour situations of optional familialism, but also, if they arise in a framework of scarce services in kind, they can reinforce a framework of implicit or explicit familiarism.^12,27,32^

 Other differences among countries are related to the level of education and the employment situation of the non-professional caregivers. Caregivers from Northern European countries (Sweden, Denmark and Finland) and also Austria have a much higher level of education than those in the rest of Europe. Luxembourg and Germany are the countries with the largest numbers of non-professional caregivers with a paid job, whereas in Albania and the FYR of Macedonia there is a large number of unemployed caregivers. Although caution is needed when interpreting these results as the level of education and employment vary across Europe. These differences might be explained by the substantially different working environments and policies on promotion in each country.^54^

 It is important to emphasize that the percentage of people under 65 years of age who currently care for dependents was almost 85% of the total non-professional caregivers identified (64.6 million people). Then, LTC policies, either to choose to favour family care, either to defamiliarise care, or to make it optional, should consider the personal conditions, not only of the people cared for but also of the carers. In this sense, these policies must take into account that a significant part of caregivers are of working age and that their needs may differ substantially from those of older carers. Thus, it is important to note that a specific measure may have different effects on different types of caregivers, in the same way that a specific policy will be conditioned by the rest of LTC policies, not only by the provision of services in kind but also by time policies – paid and unpaid leaves, working time flexibility, part time work – and cash benefits- tax deductions for purchasing professional services, monetary benefits for caregivers and cash for care.^27^

 Some issues need to be mentioned. Firstly, the opportunity cost method was the option chosen for valuing non-professional time care. The opportunity cost method is the most common technique used in the literature.^48^ So, our election facilitates comparability with studies focused on subgroups of specific caregivers, as well as it eases of access to salary data. Additionally, other methodologies (such as contingent valuation or proxy good method) are not feasible to implement due to the lack of information available for several countries. Another aspect that needs to be considered is the fact that it was not possible to identify whether the caregivers provided care for someone living at the same home or out of home. This might have reported value information about the potential differences between these two caregivers profiles. Thirdly, the shadow price to estimate the unpaid time was the minimum average wage in each country considered while other studies used other different ones.^48^ This may cause differences in terms of caregiving value across studies. Our analysis also shows the results obtained applying the median wages instead of using the average wages, but the figures do not vary significantly.

 Broadly, this paper has highlighted the fact that the role and the weight of non-professional caregivers can vary significantly among European LTC systems. However, a trend that we can clearly identify in the recent literature is the agreement that LTC should not fall exclusively on the family, and it cannot be the sole responsibility of the State either.^23,55^ In this sense, the work of Mair et al,^56^ carried out in 14 European countries, indicated that “middle-aged and older adults with chronic disease whose health limits their ability to perform paid work, who do not receive personal care from informal sources, and who live in nations with generous LTC funding, are less likely to prefer family-based care and more likely to prefer state-based care.” Given the methodology applied in the study, it cannot be inferred that social preferences have led to this result, and an inverse interpretation is also possible. This is mainly explained by the fact that in the absence of investment in LTC services, families have had to face the care of dependent people, and societies have accepted this situation as something natural.^57,58^ Perhaps both explanations are complementary, and the historical explanation is not as relevant as the prospective one: what dependent people, and their families, want today, it may be very different from what they will want in the coming decades in relation to the combination of professional and non-professional care, taking into account demographic and social changes. In any case, our data clearly indicate a high heterogeneity among European countries in relation to the contributions of non-professional care. This means that, despite the great development that LTC care systems (in terms of higher coverage, supply and quality of the services provided) have experienced and the increase in professional care resources provided to citizens in the last decades, non-professional resources far surpass professional care in several European countries.^17^

 Another trend observed in the literature is that some countries have shown in recent years a marked preference for home care over residential care. The significant process of deinstitutionalisation and emphasis on the development of home care has been observed in some Nordic countries. However, in Southern European countries the number of LTC places in long-term residential facilities have increased in recent years.^17^ This is mainly due to the incipient processes of reforming LTC systems and with the increase in the employment of women in several of these countries.^51^ The recent crisis caused by severe acute respiratory syndrome coronavirus 2 (SARS-CoV-2) could accentuate the preference for home care since long-term residential facilities’ have been an epicentre of infections in most western countries, with high mortality rates in the resident population.^59^ Additionally to these changes (in terms of the mortality and complicated situations experienced in long-term residential facilities’due to SARS-CoV-2), there are new forms of long-term residential facilities’ such as sheltered housing or houses shared by older people, and there are technological improvements adapted to homes. The latter include monitoring and communication elements, home automation adaptations,^60-65^ highlighting technological improvements adapted to homes, including both monitoring and communication elements, home automation adaptations, and technology tools.^66-70^ These improvements could mean that people who were previously admitted to long-term residential facilities now have the option of living in their own home. Although professional care and non-professional care have so far been revealed to be more substitute than complementary services,^71-73^ in a scenario of increasing preferences for receiving care at home, non-professional care and professional care could reinforce each other, being complementary. However, it will be difficult to see how this could happen without the support of non-professional care, especially when the development of home-help services is highly heterogeneous among European countries.

 A desirable transition scenario, in the medium term, where there is a strong preference for living at home, would therefore be to continue to have a certain amount of non-professional care. In those countries where we have observed that the duration of non-professional care is longer, this, as the literature warns us, translates into a greater burden for caregivers, as well as a higher prevalence of problems in the health, labour and socio-family dimensions. Its duration would therefore have to be considerably reduced.^74-78^

 In conclusion, the information provided here should help to clarify the importance of the different roles performed by family caregivers in 33 different European countries. This would help policy-makers in each country when they are designing any strategy or policy related to LTC systems. It might also lead to think that, because of the high heterogeneity among LTC systems (meaning that several of which are clearly focused on professional services while others are focused on non-professional services), it would be advisable to establish a proper balance between the two types of caregiving. In order to reduce the high opportunity cost that family caregivers burden and improve their well-being, and for buildings more solid LTC systems, governments should pay more attention to family caregiving. This would imply to promote measures to give social prestige to carers, both professionals and non-professionals, and seek to achieve an optimal combination of policies in order to obtain an appropriate balance between both types of care.

## Conclusion

 We estimated that the time of non-professional care provided in Europe reached 72 301.5 million hours in 2016. The economic value of this large amount of time is estimated, using the opportunity cost method, at 576 000 million euros, which represents approximately 3.63% of the GDP in Europe. By country, France, the United Kingdom, Italy, Spain, Turkey and Germany are those with the highest total economic cost of non-professional caregiving.

## Ethical issues

 Authors declare that ethical approval was not necessary for performing this study as it was used secondary data.

## Competing interests

 Authors declare that they have no competing interests.

## Authors’ contributions

 LMPL carried out the analysis. LMPL and JOM wrote the manuscript.

## Funding

 This work has received funding from the Ministry of Economy (ECO2017-83771-C3-1-R). The funder had no role in study design, data collection and analysis, decision to publish, or preparation of the manuscript.

## Supplementary files

Supplementary file 1 contains Tables S1 and S2.
