# Supplementary material for: The Economic Value of Non-professional Care: A Europe-Wide Analysis
Source: Int J Health Policy Manag. 2021 Oct 30;11(10):2272–86. doi: 10.34172/ijhpm.2021.149 (PMC9808255; doi:10.34172/ijhpm.2021.149)
Supplement: Supplementary file 1 — contains Tables S1 and S2. [file ijhpm-11-2272-s001.pdf]

Article title: The Economic Value of Non-professional Care: A Europe-Wide Analysis

Journal name: International Journal of Health Policy and Management (IJHPM)

Authors' information: Luz María Peña-Longobardo*, Juan Oliva-Moreno

Faculty of Law and Social Sciences, University of Castilla-La Mancha, Toledo, Spain.

(*Corresponding author: Luzmaria.pena@uclm.es)

Supplementary file 1

Table S1. Description of the variables of interest

Variables of interest	Description in European Quality of Life Survey
On average, how many hours per week are you involved in any of the following activities outside of paid work?	Caring for disabled or infirm family members, neighbours or friends under 75 years old; Caring for disabled or infirm family members, neighbours or friends aged 75 or over
High Level of education	Short-cycle tertiary education; Bachelor or equivalent; Master or equivalent; Doctoral or equivalent
Upper secondary or post-secondary education;	Upper secondary education; Post-secondary non-tertiary education
Lower secondary education or below	Early childhood Education; Primary education; Lower secondary education
Employed	At work as employee or employer/self-employed; Employed, on childcare leave; Employed, on other special leave (e.g. sickness; not holiday); In receipt of retirement pension and at work as employee or employer/self-employed; At work as relative assisting on family business or farm
Unemployed	Unemployed less than 12 months; unemployed 12 months or more
Unable to work	Unable to work due to long-term illness or disability
Retired	Retired
Full-time homemaker	Full-time homemaker / fulfilling domestic tasks
Student	In education (at school, university, etc.) / student
Other employment situation	Other (NOT ASKED/NOT ON CARD)

Table S2. Non-professional hours provided and value of non-professional care by country, with median wage. EUR 2016

Country	Annual non-professional caregiving value (total)	Annual average non-professional caregiving value (per caregiver)
Albania	482.775.392,79	1.146,33
Austria	6.152.801.439,47	9.682,18
Belgium	16.799.709.010,65	6.798,18
Bulgaria	1.241.802.501,60	2.257,05
Croatia	1.821.843.226,58	3.420,45
Cyprus	487.181.825,36	5.121,22
Czech Republic	4.202.290.750,00	3.553,09
Denmark	3.839.432.440,23	5.380,39
Estonia	373.155.740,85	2.477,20
Finland	5.201.928.206,26	4.981,31
France	126.529.636.048,64	8.212,82
FYR of Macedonia	323.868.458,56	2.066,23
Germany	35.858.917.110,15	6.863,93
Greece	6.107.364.856,43	5.033,54
Hungary	2.458.889.396,67	3.177,88
Ireland	7.773.221.890,07	13.208,04
Italy	48.659.438.464,09	6.113,21
Latvia	1.117.305.081,28	2.681,46
Lithuania	1.103.012.354,00	3.282,62
Luxembourg	1.053.366.004,16	10.767,97
Malta	449.141.001,03	6.510,04
Montenegro	292.004.994,76	4.088,45

Netherlands	17.049.310.118,59	6.180,74
Poland	20.523.825.929,29	5.120,86
Portugal	2.095.809.631,96	3.494,82
Romania	7.894.643.212,24	3.067,16
Serbia	2.497.087.380,61	2.009,49
Slovakia	1.990.993.871,99	4.080,75
Slovenia	1.530.652.340,17	5.610,63
Spain	42.360.708.625,61	6.960,33
Sweden	4.939.179.119,96	5.058,46
Turkey	44.667.178.890,06	5.767,97
United Kingdom	98.457.174.845,55	10.573,05
**TOTAL**	**516.335.650.159,66**	**6.778,51**

Source: Own elaboration
